# TLR/MyD88-Mediated Inflammation Induced in Porcine Intestinal Epithelial Cells by Ochratoxin A Affects Intestinal Barrier Function

**DOI:** 10.3390/toxics11050437

**Published:** 2023-05-06

**Authors:** Jung Woong Yoon, Sangsu Shin, JeongWoong Park, Bo Ram Lee, Sang In Lee

**Affiliations:** 1 Department of Animal Science and Biotechnology, Kyungpook National University, Sangju-si 37224, Republic of Korea; kizx789@knu.ac.kr (J.W.Y.); sss@knu.ac.kr (S.S.); supermantm01@naver.com (J.P.); 2 Research Center for Horse Industry, Kyungpook National University, Sangju-si 37224, Republic of Korea; 3 Department of Animal Biotechnology, Kyungpook National University, Sangju-si 37224, Republic of Korea; 4 Animal Biotechnology Division, National Institute of Animal Science, Rural Development Administration, Wanju-gun 55365, Republic of Korea

**Keywords:** ochratoxin A, IPEC-J2 cells, TLR/MyD88 signaling, tight junction, intestinal barrier, inflammation

## Abstract

The intestinal epithelium performs vital functions such as nutrient absorption and acting as an intestinal barrier to maintain the host’s homeostasis. Mycotoxin, which affects the processing and storage of animal feedstuff, is a problematic pollutant in farming products. Ochratoxin A generated by *Aspergillus* and *Penicillium* fungi causes inflammation, intestinal dysfunction, decline in growth, and reduced intake in porcine and other livestock. Despite these ongoing problems, OTA-related studies in intestinal epithelium are lacking. This study aimed to demonstrate that OTA regulates TLR/MyD88 signaling in IPEC-J2 cells and induces barrier function impairment through tight junction reduction. We measured expression of TLR/MyD88 signaling-related mRNAs and proteins. The indicator of intestinal barrier integrity was confirmed through immunofluorescence and transepithelial electrical resistance. Additionally, we confirmed whether inflammatory cytokines and barrier function were affected by MyD88 inhibition. MyD88 inhibition alleviated inflammatory cytokine levels, tight junction reduction, and damage to barrier function due to OTA. These results indicate that OTA induces TLR/MyD88 signaling-related genes and impairs tight junctions and intestinal barrier function in IPEC-J2 cells. MyD88 regulation in OTA-treated IPEC-J2 cells mitigates the tight junction and intestinal barrier function impairments. Our findings provide a molecular understanding of OTA toxicity in porcine intestinal epithelial cells.

## 1. Introduction

Intestinal epithelial cells contribute to nutrient uptake and form the luminal barrier boundary against harmful factors for the host [1,2]. The intestinal epithelium, organized by epithelial cells, is a vital mediator of the host responsible for necessary physiological maintenance, such as transporting nutrients into the body and the protection from harmful factors [3,4]. Epithelial homeostasis is typically maintained via safe and fresh food uptake and is vulnerable to ingestion of contaminated foods or oral invasion of pathogens and natural toxins [5]. Epithelial cells damaged by contaminants and different environments cause morphological changes and tight junction disruption, resulting in increased paracellular transport and intestinal barrier dysfunction [6,7]. Increased luminal permeability can affect the critical functions of the small intestine, such as immune response and prevention of external factor transmission [8,9]. Therefore, maintaining small intestinal barrier function is essential for the physiological balance of the host.

Ochratoxin A (OTA), one of the harmful mycotoxins, is a worldwide problem as the secondary metabolite generated by *Aspergillus* and *Penicillium* fungi. OTA flourishes in moderate environments and industrial processes, including tropical temperatures and humidity and food product processing facilities [10,11,12]. OTA is a global problem and can contaminate diverse food and feedstuffs, such as grains, vegetables, forages, and manufactured products [13,14]. Ingestion of contaminants can lead to diverse toxic effects with both acute and chronic diseases affecting the health of humans and livestock, resulting in a major economic burden [15,16]. Several studies have shown that OTA exposure occurs in various organs and targets the livestock industry, particularly in chickens and pigs [17,18]. Recently, OTA-mediated toxic effects on the intestinal epithelium have been reported. The intestinal epithelium is the potential target of OTA exposure because it plays a role as the barrier to external factors [19,20]. In diverse animal research, it has demonstrated that OTA toxicity impacts the intestinal epithelium and causes intestinal barrier dysfunction [21,22]. Nevertheless, studies on the mechanism of OTA’s effects on the intestinal barrier are lacking.

Toll-like receptors (TLRs) are one of the pattern recognition receptors that detect various external elements. It is located in the cell membrane and cytoplasm and is regulated in various ways by pathogen-associated molecular patterns (PAMPs) present in extrinsic elements. TLRs are activated and act as the defense interface of the host in innate and adaptive immunity when PAMPs are detected [23]. Activation of TLRs induces myeloid differentiation protein 88 (MyD88) which is a fundamental adaptor protein in the TLR signaling pathway except for TLR3. MyD88 activates various immune cells via TLR signaling, thereby serving as an immune response and host defense [24]. Although MyD88 is distributed in several tissues, it is mainly expressed in the small intestine, as it is the first barrier to foreign factors [25,26]. Thus, we surmise that alteration of the intestinal barrier by exposure to OTA may occur through MyD88 induction.

This study examined the correlation between the intestinal barrier and MyD88 through IPEC-J2, intestinal epithelial cells derived from neonatal porcine jejunum. Additionally, we investigated the genes associated with MyD88 after OTA treatment.

## 2. Materials and Methods

### 2.1. Cell Culture and Treatment

The porcine intestinal cells (IPEC-J2) were cultured in media that contained 90% Dulbecco’s Modified Eagle Medium (DMEM) (Thermo Fisher Scientific, Waltham, MA, USA), 10% fetal bovine serum (FBS), and 1% penicillin-streptomycin (PS) and incubated in incubators with 5% CO_2_ and 37 °C. Ochratoxin A (OTA; Sigma-Aldrich, St. Louis, MO, USA) and MyD88 inhibitor (TJ-M2010-5, MedChemExpress, Monmouth Junction, NJ, USA) were diluted in dimethyl sulfoxide (DMSO), and each solution was diluted in media before treated cells. The OTA treatment protocol was the same as in our previous study, with 4 μM OTA for 48 h [27].

### 2.2. qRT-PCR and Western Blotting

For RNA sample preparation, cells were seeded at a concentration of 1 × 10^5^ in 6-well plates. The cells were cultured until they reached 70–80% confluency and were treated with 4 μM OTA for 48 h. Additionally, to collect the sample cells, they were treated with 0.25 trypsin-EDTA for 5 min, and RNA was extracted using the AccuPreP Universal RNA extraction kit (Bioneer, Daejeon, Republic of Korea). Total RNA was quantified using a P200 Micro-volume spectrophotometer (Biosis Design, Gwangmyeong-si, Republic of Korea). To ascertain the mRNA expression level, 1 μg RNA was used as a first-strand cDNA synthesis template using the DiaStar RT Kit (SolGent, Daejeon, Republic of Korea). The primers for target gene amplification used in qRT-PCR were designed using the Primer 3 program (http://frodo.wi.mit.edu). qRT-PCR under specific reaction conditions (95 °C for 3 min, 40 cycles of 95 °C for 15 s, 56–60 °C for 15 s, and 72 °C for 15 s) was performed using a 7500 Fast Real-Time PCR system. Finally, the mRNA expression levels were normalized to those of the control (GAPDH; glyceraldehyde-3-phosphate dehydrogenase). The qRT-PCR results were analyzed using the 2^−ΔΔCT^ method: ΔCt = Cq (treated) − Cq (control) and ΔΔCt = ΔCt (treated) − ΔCt (control). Table 1 lists the sequences of the primers used in this study.

For protein sample preparation, cells were seeded at concentration of 1 × 10^5^ in 6-well plates. The cells were cultured for 48 h to reach 70–80% confluency and then treated with 4 μM OTA for 48 h. IPEC-J2 cells were rinsed with phosphate-buffered saline (PBS) and treated with 1× lysis buffer (Cell signaling Technology, Danvers, MA, USA) containing Protease Inhibitor Cocktail (DAWINBIO, Hanam-si, Republic of Korea) on ice for 5 min. The cells were scraped using a cell scraper. The protein samples were collected and vortexed for 5 min. The samples were then centrifuged for 10 min at 14,000× *g* at 4 °C. After 10 min, the supernatant was carefully collected to ensure no precipitate was collected. The protein supernatant was quantified using the Pierce BCA Protein Assay Kit (Thermo Fisher Scientific). An equal volume of 2× Laemmli buffer (4% sodium dodecyl sulfate, 10% 2-mercaptoethanol, 20% glycerol, 0.004% bromophenol blue, and 0.125 M Tris-HCl) was added to the protein sample (10 μg) and denatured at 95 °C for 5 min. The protein sample was separated via electrophoresis on a 9% SDS-PAGE gel for 1 h at 120 V and transferred onto PVDF membranes. The membranes were washed thrice with TBST (20 mM Tris, pH 7.5; 150 mM NaCl; and 0.1% Tween 20) and blocked with a blocking buffer (5% skim milk in PBS) for 1 h. After blocking, the membranes were incubated with primary antibodies (β-actin, 1:2000, Santa Cruz Biotechnology, Dallas, TX, USA; MyD88, 1:1000, MybioSouce, San Diego, CA, USA), which were diluted in 5% skim milk overnight at 4 °C. After primary antibody treatment, the membranes were washed thrice with TBST and incubated with secondary antibodies, which were diluted in 2% skim milk for 1 h at room temperature. The immunocomplex was visualized using an ECL Western blot substrate and imaged using the ChemiDoc imaging system. Each protein band was quantified using Image J.

### 2.3. Immunofluorescence

A Microscope Cover Glass 10 mm (Labpia, Seoul, Republic of Korea) was placed in each well of 24-well plates and coated using a 0.1% gelatin solution (Sigma Aldrich). The plate was incubated at 37 °C for 1 h and the remaining solution was aspirated. For immunofluorescence, IPEC-J2 cells were seeded onto coverslips coated with 0.1% gelatin. The cells were fixed with 4% paraformaldehyde (degree of polymerization of 8–100 units) (Biosolution, Seoul, Republic of Korea) and blocked with a blocking buffer. After blocking, the cells were treated with primary antibodies (ZO-1, 1:200, Thermo Fisher Scientific), which were diluted with an antibody buffer overnight at 4 °C. The cells were washed with PBS three times and were treated with an anti-rabbit IgG secondary antibody (Alexa fluor 488) (Thermo Fisher Scientific, Carlsbad, CA, USA) for 1 h at room temperature in the dark. Finally, the coverslips with cells were mounted using VECTASHIELD Antifade Mounting Medium with DAPI (Vector Laboratories, Burlingame, CA, USA) and then imaged using an inverted fluorescence microscope (Korealabtech, Seongnam-si, Republic of Korea).

### 2.4. Transepithelial Electrical Resistance (TEER)

IPEC-J2 cells were seeded into a 24-well hanging transwell chamber (polycarbonate membrane, pore size 0.4 μm, surface area 0.33 cm^2^, SPL) at a concentration of 2 × 10^4^ cells and grown to 70–90% confluency. The cells were treated with or without OTA and TJ-M2010-5 for 48 h when the cells reached 70–90% monolayer confluence. The cells were washed twice with PBS and transferred to the upper and lower chambers. For TEER assessment, the upper and lower chambers were treated with PBS. TEER was assessed using an epithelial volt/ohm meter (World Precision Instruments, Sarasota, FL, USA). Prior to measurement, the electrodes were treated with isopropyl alcohol (Biosesang, Yongin-si, Republic of Korea) for 10 min and rinsed with PBS. Long and short electrodes were measured perpendicular to the upper and lower chamber, respectively. TEER values were calculated by subtracting the value for the blank chamber from the raw value for each chamber and multiplying by the surface area.

### 2.5. Statistical Analysis

All experiments were performed independently three times. Significant differences between treatments were analyzed using a general linear model (PROC-GLM) procedure in SAS. In graphs, the data are presented as means and standard errors. Statistical data comparisons were analyzed using Student’s *t*-test and GLM, and *p*-values lower than 0.05 were considered statistically significant. Significant differences between treatment groups were evaluated using Duncan’s multiple range tests.

## 3. Results

### 3.1. OTA Exposure Affects TLR Expression in IPEC-J2 Cells

**Figure 1:**
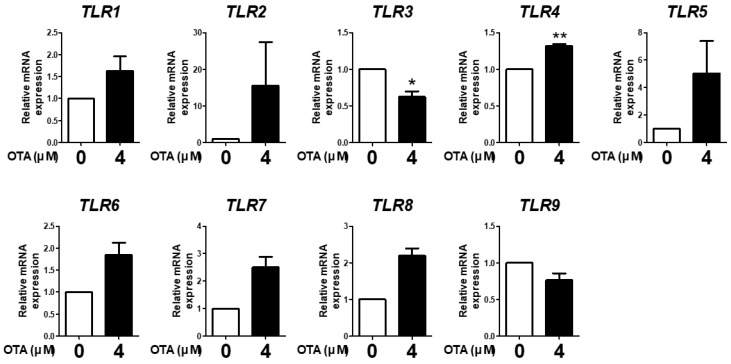
Ochratoxin A affects Toll-like receptor signaling in IPEC-J2 cells. The relative mRNA levels of Toll-like receptor genes (*TLR1*, *TLR2*, *TLR3*, *TLR4*, *TLR5*, *TLR6*, *TLR7*, *TLR8*, and *TLR9*) after treatment with 4 μM OTA4 compared with the untreated group. * *p* < 0.05 and ** *p* < 0.01 indicate significant differences between control and treatment groups. Each error bar represents the standard errors (sEs) of the analysis, which was conducted in triplicate.

We evaluated the expression of *TLR1*, *TLR2*, *TLR3*, *TLR4*, *TLR5*, *TLR6*, *TLR7*, *TLR8*, and *TLR9* in IPEC-J2 cells treated with 4 μM OTA for 48 h to analyze whether OTA induced TLR expression. The results showed that OTA treatment significantly decreased *TLR3* mRNA expression and increased *TLR4* mRNA expression compared with the control. There were no statistically significant differences in the other TLRs; however, *TLR1*, *TLR2*, *TLR5*, *TLR6*, *TLR7*, and *TLR8* expression showed an increasing trend, whereas *TLR9* expression showed a decreasing trend (Figure 1). These results indicate that OTA treatment altered TLR expression in IPEC-J2 cells.

### 3.2. OTA Exposure Induces MyD88 Activation

**Figure 2:**
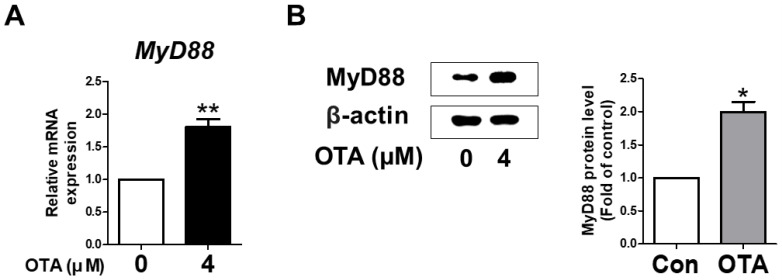
Ochratoxin A regulates the expression of myeloid differentiation primary response 88 (MyD88). (**A**) The relative mRNA level of MyD88 gene treated with 4 μM OTA compared with untreated group. (**B**) The protein level of MyD88 was measured via Western blotting. The protein level is shown as quantitative data compared with the control. The protein bands of β-actin and MyD88 were quantified through Image J software. * *p* < 0.05 and ** *p* < 0.01 indicate significant differences between the control and treatment groups. Each error bar represents the standard errors (sEs) of the analysis, which was conducted in triplicate.

To verify whether MyD88 activation in IPEC-J2 cells was induced upon treatment with OTA, the levels of mRNA and protein were measured using qRT-PCR and Western blotting. As expected, in IPEC-J2 cells treated with OTA, MyD88 mRNA expression was significantly higher than in the untreated group. In addition, Western blot analysis showed a significant increase in the protein level of MyD88 with OTA treatment compared to the control group (Figure 2B). These results indicate that OTA treatment induced MyD88 activation in IPEC-J2 cells.

### 3.3. OTA Exposure Is Related to Downstream TLR/MyD88 Signaling

**Figure 3:**
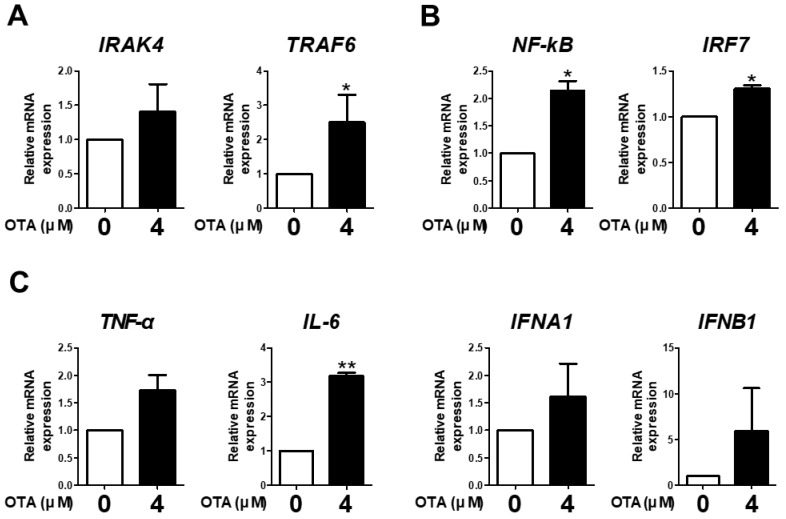
Ochratoxin A is related to downstream TLR signaling in IPEC-J2 cells. (**A**) The relative mRNA levels of *IRAK4* and *TRAF6* genes after treatment with 4 μM OTA compared with the control group. (**B**) The relative mRNA levels of transcription factor (*NF-kB* and *IRF7*) genes after treatment with 4 μM OTA compared with the untreated group. (**C**) The relative mRNA levels of cytokine (*TNF-α*, *IL-6*, *IFNA1*, and *IFNB1*) genes in cells treated with 4 μM OTA compared with the control group. * *p* < 0.05 and ** *p* < 0.01 indicate significant differences between control and treatment groups. Each error bar represents the standard errors (sEs) of the analysis, which was conducted in triplicate.

The expression of *IRAK4*, *TRAF6*, *NF-kB*, *IRF7*, *TNF-α*, *IL-6*, *IFNA1*, and *IFNB1* was measured to examine the effect of OTA treatment on TLR/MyD88 signaling. OTA treatment significantly increased the mRNA expression of *TRAF6* compared with the control (Figure 3A). We found a significant increase in the mRNA expression of *NF-kB* and *IRF7* with OTA treatment (Figure 3B). Additionally, OTA treatment significantly increased the mRNA expression of *IL-6* in IPEC-J2 cells (Figure 3C). Although there were no significant differences in the expression of *IRAK4*, *TNF-α*, *IFNA1*, and *IFNB1*, there were overall increasing trends. Therefore, we surmised that OTA treatment was associated with TLR/MyD88 signaling in IPEC-J2 cells.

### 3.4. OTA Exposure Causes Tight Junction Disruption and Intestinal Barrier Damage

**Figure 4:**
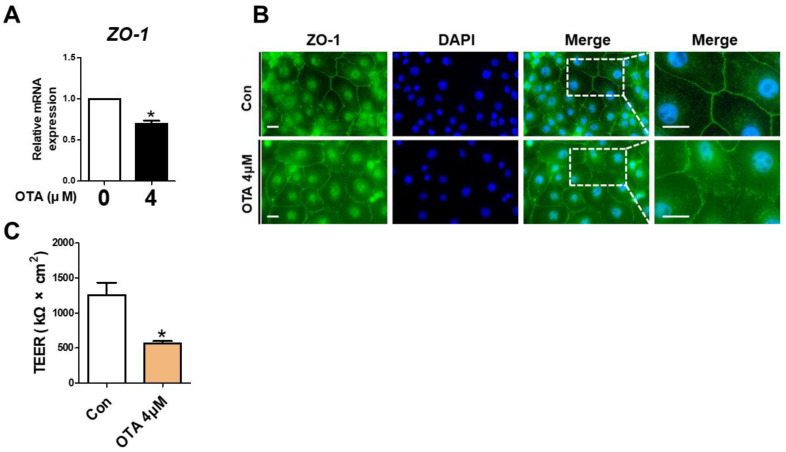
Ochratoxin A damages intestinal barrier function. (**A**) The relative mRNA level of ZO-1 gene in cells treated with 4 μM OTA compared with untreated group. (**B**) Immunocytochemistry of tight junction ZO-1 in control cell and treatment cell groups. Blue dye indicates nuclei stained with 4′,6-diamidino-2-phenylindole (DAPI), and green dye indicates ZO-1 stained with Alexa fluor 488. Scale bar is 40 μm. (**C**) Analysis of TEER in IPEC-J2 cells treated with OTA. * *p* < 0.05 indicates a significant difference between control and treatment groups. Each error bar represents the standard errors (sEs) of the analysis, which was conducted in triplicate.

To assess the effect of OTA treatment on tight junctions, *ZO-1* expression was determined using RT-qPCR and immunofluorescence. Compared to the control, the *ZO-1* mRNA level exhibited a significant decline in OTA-treated IPEC-J2 cells (Figure 4A). As shown in Figure 4B, compared with the control group, a considerable disruption was noted in the *ZO-1* contour in the OTA-treated groups. Next, we determined whether OTA treatment affects intestinal barrier function through TEER, which generally indicates epithelial competence. As expected, the TEER value was significantly lower in OTA-treated IPEC-J2 cells than in control cells (Figure 4C). These results suggested that OTA treatment can induce tight junction disruption and intestinal barrier damage in IPEC-J2 cells.

### 3.5. MYD88 Inhibition Alleviates Proinflammatory Cytokine and Intestinal Barrier in OTA Exposure

**Figure 5:**
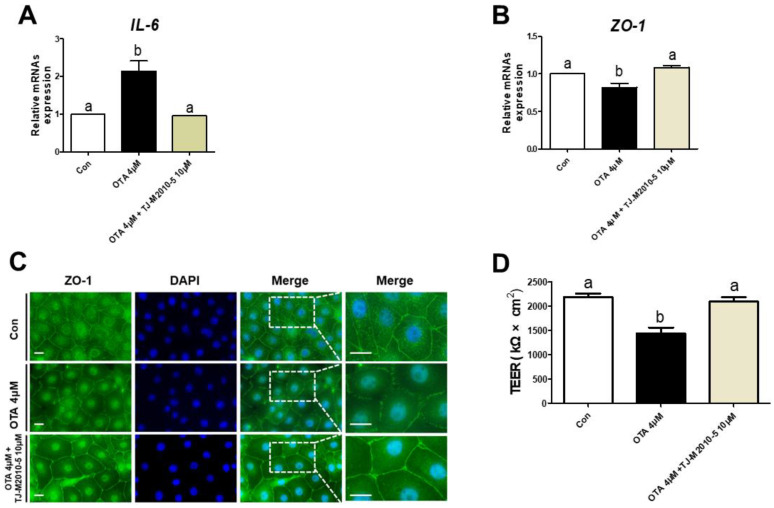
MyD88 inhibition alleviated the detrimental response to ochratoxin A. In IPEC-J2 cells treated with OTA or TJ-M2010-5, expression of (**A**) IL-6 and (**B**) ZO-1 mRNA was measured using RT–qPCR. (**C**) Immunocytochemistry of tight junction ZO-1 in control, OTA treatment, and OTA and MyD88 inhibition (TJ-M2010-5) co-treatment cell groups. Blue dye indicates nuclei stained with DAPI, and green dye indicates ZO-1 stained with Alexa fluor 488. Scale bar is 40 μm. (**D**) TEER values were estimated in IPEC-J2 cells treated with OTA or TJ-M2010-5. Significant differences between each group are represented by a and b. Each error bar represents the standard errors (sEs) of the analysis, which was conducted in triplicate.

We treated IPEC-J2 cells with or without OTA or TJ-M2010-5, a MYD88 inhibitor, to investigate the correlation between TLR/MyD88 signaling and intestinal barrier function. The OTA treatment significantly increased the mRNA expression of *IL-6*, and the IPEC-J2 cells treated with OTA and TJ-M2010-5 were statistically identical to the control (Figure 5A). Next, we confirmed whether TJ-M2010-5 affected the expression of *ZO-1* after OTA treatment. The OTA treatment showed significant inhibition of *ZO-1*, as shown in Figure 5B,C. However, co-treatment with OTA and TJ-M2010-5 maintained the expression of *ZO-1* in IPEC-J2 cells. Furthermore, the decrease in the TEER value due to OTA treatment was preserved through TJ-M2010-5 treatment (Figure 5D).

## 4. Discussion

The intestinal epithelium plays specific roles, such as the transport of nutrients and defense against contaminant factors, as a vital component of homeostasis of the host [1,3]. The intestinal epithelium is easily targeted by external substances and in animal growth, the intestinal epithelium is an important organ in which physiological and demanding events frequently occur. If persistent damage induces intestinal dysfunction, there will be reduced feed intake and growth, and potential intestinal disease [8,17]. Therefore, supplying sanitary feed is necessary for the appropriate functioning of the intestinal epithelium. Mycotoxin is a secondary metabolite generated by various fungi and has been detected in corn and grains, the main raw materials for livestock feed [28]. Among the mycotoxins, OTA, which has spread worldwide, causes contamination of diverse food and feedstuffs. These contaminants enter the body orally and have adverse effects, such as reduced food intake, weight loss, and barrier and immune dysfunction [10,12,29,30]. As the OTA-mediated problems gradually increase, the intestinal epithelium acts as the first interaction site in the body, so it is more vulnerable than other organs and causes economic losses in the livestock industry [16,17,18,31]. Although several studies have shown that OTA exposure affects the intestinal barrier and immune function, the mechanism of OTA-mediated intestinal impairment needs to be investigated in detail.

We used intestinal porcine cells (IPEC-J2) as the in vitro model. First, we determined whether OTA exposure affected TLRs. TLRs act as a detector in the immune system to recognize invading microorganisms, such as Gram-positive and Gram-negative bacteria, viruses, and fungi [23]. We confirmed that OTA induces a significant decline in TLR3 expression and significant increase in TLR4 expression in IPEC-J2 cells. Although OTA did not induce significant alterations in the expression of the other TLRs, the overall results showed an increasing trend. We then evaluated the adaptor protein that is the recognition site of TLR signaling and efficiently responds depending on the microorganism [23]. We selected MyD88 among several adaptor candidates, including MyD88, Mal, TRIF, and TRAM. MyD88 is located on both the cell surface and endosomes and is especially associated with all TLRs, except for TLR3 [32]. We revealed that OTA treatment markedly decreased TLR3 expression in IPEC-J2 cells. Furthermore, OTA significantly enhanced MyD88 activation in IPEC-J2 cells. These results suggest that OTA is involved in TLR/MyD88 signaling.

TLR/MyD88 signaling recruits IRAK4, which plays an essential role in signaling activation and binds to TRAF6 to accelerate the signaling. These stimulations lead to the activation of IRF7 and NF-κB, which are transcription factors crucial to subsequent signaling [33,34]. After the activation of these factors, IRF7 induces the activation of IFNs, such as IFNA and IFNB, and NF-κB regulates the expression of proinflammatory cytokine genes [23,35]. We ascertained that OTA exposure was correlated with TLR/MyD88 signaling in IPEC-J2 cells and subsequently analyzed the expression of TLR/MyD88-related genes. Our study revealed that OTA exposure increased *TRAF6*, *NF-κB*, *IRF7*, and *IL-6* expression. Although OTA did not significantly increase the expression of the other genes, the overall results indicated an increasing trend. This finding further supports the correlation between OTA and TLR/MyD88 signaling in IPEC-J2 cells.

The intestinal barrier is structurally maintained by cell–cell adhesions, including gap junctions, desmosomes, adherens junctions, and tight junctions, which can be organized between epithelia [36]. Among these adhesions, the formation of tight junctions regulates paracellular passage, i.e., the movement of extrinsic or intrinsic elements, such as solutes, ions, and pathogens, from the lumen to the lamina propria [37,38]. Tight junctions have a dynamic and complex structure that endured constant stimulation in the intestinal epithelium. These are composed of several transmembrane proteins, such as zonula-1, claudins, and occludin [39,40]. The role of these proteins is not fully understood, but many studies suggest that they play an important role in normal intestinal barrier function.

Previous studies have revealed that diverse factors induce inflammation-mediated intestinal barrier impairment in IPEC-J2 cells [41,42,43]. The intestinal integrity is preserved by tight junctions, which block paracellular movement of noxious factors and are necessary to form the intestinal barrier. We found that OTA could induce inflammation because it activates the expression of various inflammation-related genes. To verify the intestinal barrier function, the expression and distribution of tight junctions and TEER values were investigated in OTA-induced IPEC-J2 cells. As expected, OTA treatment reduced tight junction expression and distribution and decreased TEER values in IPEC-J2 cells.

OTA-induced intestinal barrier dysfunction may be associated with MyD88 activation in IPEC-J2 cells. MyD88 is an adaptor protein that plays a central role in various inflammatory responses and activates signaling pathways through pattern recognition receptors located on the cell surface or endosomes. MyD88 activation leads to the production of proinflammatory cytokines and type I interferons [24,25,26]. Additionally, the activation of various inflammatory cytokines has been indicated to inhibit tight junctions and intestinal barriers [44,45]. Thus, we used TJ-M2010-5, an MyD88 inhibitor, to disrupt TLR/MyD88 signaling. In this study, we demonstrated that the levels of the proinflammatory cytokine IL-6 increased and that OTA disrupted tight junctions and the intestinal barrier in IPEC-J2 cells. Our results showed that TJ-M2010-5 alleviated the levels of OTA-induced inflammatory cytokines. We also revealed that TJ-M2010-5 mitigated OTA-mediated impairment of tight junction proteins and the intestinal barrier.

## 5. Conclusions

**Figure 6:**
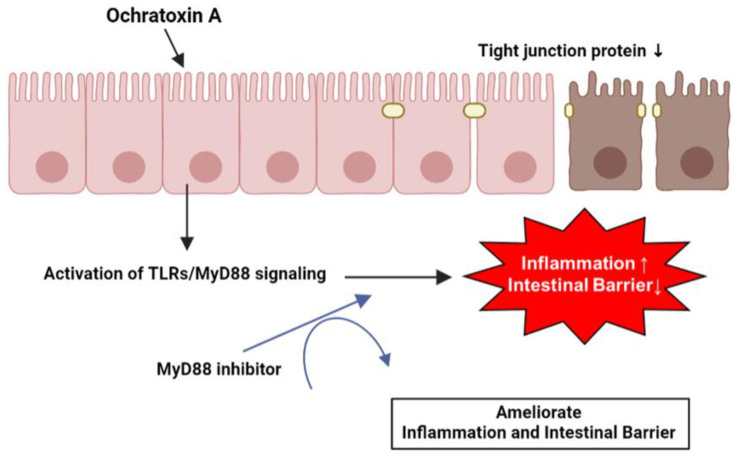
Schematic illustration of the proposed mechanism for OTA-mediated inflammation and intestinal barrier dysfunction. OTA affects inflammation and intestinal barrier function via TLR/MyD88 signaling and Myd88 inhibition attenuated OTA-mediated inflammation and intestinal barrier dysfunction in IPEC-J2 cells.

OTA induces the expression of various inflammation-related genes by activating the TLR/MyD88 signaling in IPEC-J2 cells. Likewise, OTA directly disrupts tight junction proteins and the intestinal barrier in IPEC-J2 cells. Furthermore, the increase in inflammatory cytokine levels and reduction in tight junction proteins and intestinal barrier function are facilitated by MyD88 regulation (Figure 6). These results may provide an understanding of OTA exposure in porcine small intestinal epithelial cells and essential clues for discovering biomaterials that can alleviate OTA toxicity.

## Figures and Tables

**Table 1:** List of primers used in this study.

*Genes*	Description	Accession No.		Sequence (5′-3′)
*GAPDH*	*Glyceraldehyde-3-phosphate dehydrogenase*	NM_001206359	Forward Reverse	ACACCGAGCATCTCCTGACT GACGAGGCAGGTCTCCCTAA
*TLR1*	*Toll-like receptor 1*	NM_001031775	Forward Reverse	TGTGTTGGAAGAGGTCAGGA TGGCAAAATGGAAGATGCTA
*TLR2*	*Toll-like receptor 2*	NM_213761	Forward Reverse	CCAGGCAAGTGGATTATTGA AAGAGACGGAAGTGGGAGAA
*TLR3*	*Toll-like receptor 3*	NM_001097444	Forward Reverse	GTGCCCCATTTGAACTCTTT TGCTCTGGCTGCTTGTCTAT
*TLR4*	*Toll-like receptor 4*	NM_001113039	Forward Reverse	TGGTCAGCCTCCAAACCTTC GGAATGAAAATGCCCTCTGGG
*TLR5*	*Toll-like receptor 5*	NM_001348771	Forward Reverse	GGCTTCTGTTGGGATGTTTT CGAGGTGAGCAGGTAAGTCA
*TLR6*	*Toll-like receptor 6*	NM_213760	Forward Reverse	AGACACGGGCTGGACTTACT GCCACCTCATTTACCTCTGG
*TLR7*	*Toll-like receptor 7*	NM_001097434	Forward Reverse	CCTTCTTCCTCCTTGCCTCT TTGGCTGATGCTATTTCCTG
*TLR8*	*Toll-like receptor 8*	NM_214187	Forward Reverse	CTTCCCACATCCCAGACTTT GTCCCTCTCCTCCAAACAGA
*TLR9*	*Toll-like receptor 9*	NM_213958	Forward Reverse	TCTGACTTCGTCCACCTGTC GGTCGTGATGCTGTTGTAGC
*MyD88*	*Myeloid differentiation primary response 88*	NM_001099923	Forward Reverse	GTCGGATGGTAGTGGTTGTCT TGCTGGGGAACTCTTTCTTC
*IRAK4*	*Interleukin 1 receptor-associated kinase 4*	NM_001112693	Forward Reverse	GAACACAACTCTATGCCACCA AATCCTCCCTCTCCCATCTT
*TRAF6*	*TNF receptor-associated factor 6*	NM_001105286	Forward Reverse	TCTCTGACGGTGAAGTGTCC AAGTTGGCATTTTTGGAAGG
*NF-κB*	*Nuclear factor of kappa light polypeptide gene enhancer in B-cells 1*	NM_001048232	Forward Reverse	TCTCTGACGGTGAAGTGTCC AAGTTGGCATTTTTGGAAGG
*IRF7*	*Interferon regulatory factor 7*	XM_021076250	Forward Reverse	CGTCCTGGTGAAGTTGGAG GCTCGTCATAGAGGCTGTTG
*IL-6*	*Interleukin-6*	NM_001252429	Forward Reverse	GCTTCCAATCTGGGTTCAAT ATTCTTTCCCTTTTGCCTCA
*TNFα*	*Tumor necrosis factor* *α*	NM_214022	Forward Reverse	TTTCTGTGAAAACGGAGCTG CAGCGATGTAGCGACAAGTT
*IFNA1*	*Interferon alpha 1*	NM_214393	Forward Reverse	GGACCACAGAAGGGACTTTG CTCTCATTCCAGGCAGCAG
*IFNB1*	*Interferon beta 1*	NM_001003923	Forward Reverse	TACCAACAAAGGAGCAGCAA GGTTTCATTCCAGCCAGTG
*ZO-1*	*Zonula occludens 1*	XM_021098856	Forward Reverse	GATCCTGACCCGGTGTCTGA TTGGTGGGTTTGGTGGGTTG

## Data Availability

Not applicable.
